# Supplementary material for: Association of personal and professional factors, resilience and quality of life of registered nurses in a university medical city in the Kingdom of Saudi Arabia
Source: PLoS One. 2024 Sep 10;19(9):e0310263. doi: 10.1371/journal.pone.0310263 (PMC11386420; doi:10.1371/journal.pone.0310263)
Supplement: Attachment — Submitted filename: QOL_MH.docx [file pone.0310263.s002.docx]

**Cross-Sectional Design Limitations**

The use of a cross-sectional design limits the ability to infer causality between resilience and quality of life (QOL). While it is acknowledged that longitudinal designs may be more challenging to implement, it is important to clearly state this limitation and discuss how it affects the interpretation of the results. Additionally, the authors should consider suggesting longitudinal studies in the future research section to provide stronger evidence for causality.

**Convenience Sampling Bias**

The use of convenience sampling raises concerns about the representativeness of the sample. This method can introduce selection bias, limiting the generalizability of the findings. The authors should acknowledge this limitation more explicitly in the manuscript and discuss its potential impact on the study's conclusions.

**Self-Reported Data Concerns**

Reliance on self-reported data can introduce response biases, such as social desirability bias and recall bias. The manuscript should discuss these limitations and consider suggesting the use of objective measures or triangulation with other data sources in future research to enhance the validity of the findings.

**Lack of Clear Topic Sentences**

Several paragraphs lack clear and concise topic sentences, making it difficult for readers to follow the logical flow of the manuscript. Each paragraph should begin with a strong topic sentence that clearly introduces the main idea. The authors should revise these sections to improve readability and coherence.

**Imprecise Reporting of Statistical Results**

The statistical results reported in this study are sometimes imprecise. For example, stating the exact p-values (e.g. p = 0.032) instead of "<.05" would enhance transparency. Additionally, some tables lack sufficient details regarding the statistical tests used, making it challenging to fully interpret the results. The authors should provide more comprehensive details in the tables and the text.

**Ethical Considerations and Anonymity**

While the manuscript mentions that written informed consent was obtained, details on measures taken to ensure the anonymity and confidentiality of participants' data are also included. This information is crucial for maintaining ethical standards, and should be explicitly stated in the manuscript.

**Risks to Participants**

Potential risks to the participants were not adequately discussed. A thorough explanation of any risks involved in participation along with measures taken to mitigate these risks should be included to ensure adherence to ethical standards.

**Validation of Measurement Tools**

The manuscript mentions the validity and reliability of the Resilience Scale for Nurses and WHOQOL-BREF but lacks specific details on how these were confirmed in the current study population. Providing detailed psychometric properties specific to this sample would enhance the credibility of the instrument used.

**Translation Process**

The translation process for any non-English version of the questionnaire used was not described. This is a critical omission because ensuring the validity of the translated instruments is essential. The authors should include a description of the translation and validation process.

**Linking Discussion to Research Questions**

There are instances in which the discussion is not clearly linked to the study's aims and hypotheses. For example, the exploration of gender differences in QOL should be more explicitly connected to the research questions posed at the beginning of the study. The authors should ensure that all discussions are tied to the initial research objectives.

**Justification for Predictor Variables**

The rationale for including specific predictor variables in the regression models is inadequately justified. The manuscript should provide a clear and compelling rationale for the inclusion of each predictor variable, as supported by relevant literature.

**Global Significance of Findings**

While the focus on the Saudi Arabian context is justified, this manuscript should better articulate the global significance of the findings. Highlighting the broader implications and potential applications of the results would strengthen the contribution of the manuscript to the field.

**Detailed Justification of Hypotheses**

The introduction lacks detailed justification for the specific hypotheses tested. A more thorough explanation of the theoretical underpinnings and the empirical evidence guiding the hypotheses would provide a stronger foundation for this study.

**Discussion of Limitations**

The Limitations section is too brief and does not adequately discuss how each limitation impacts the findings. A more detailed analysis of the study's limitations and suggestions for mitigating these issues in future research are necessary.

**Specific Future Research Recommendations**

Recommendations for future research are generally general and lack specificity. Providing targeted and actionable suggestions based on the identified gaps and limitations is valuable.

**Assumptions of Multivariable Multiple Regression**

The manuscript uses multivariable multiple regression appropriately, but lacks details on the assumptions checked before performing these analyses (e.g. multicollinearity and normality). Providing this information is crucial to assess the robustness of the statistical methods used.

**Avoiding "Impact" in the Title**

The use of the term "impact" in the title is misleading, as the study's design does not allow causality to be established. A more accurate term would be "association" or "relationship.

**Articulating Study's Importance**

While this study addresses a significant topic, its importance could be better articulated in terms of its potential influence on policy and practice. The manuscript should emphasise how the findings can inform healthcare policies and interventions in more detail.

**Recommendation:**

There are substantial areas that require improvement, particularly in methodology, reporting, and discussion. Therefore, we recommend that the manuscript be revised with major changes before it can be considered for publication. These revisions should address methodological weaknesses, enhance the clarity and coherence of the writing, and provide a more robust discussion and justification of the findings.
