# Supplementary material for: Association of personal and professional factors, resilience and quality of life of registered nurses in a university medical city in the Kingdom of Saudi Arabia
Source: PLoS One. 2024 Sep 10;19(9):e0310263. doi: 10.1371/journal.pone.0310263 (PMC11386420; doi:10.1371/journal.pone.0310263)
Supplement: Attachment — Submitted filename: Response to Reviewers_PlosOne_Alshehry_V1.docx [file pone.0310263.s003.docx]

**Point-by-point response to reviewers’ comments**

**Journal Requirements:**

1. When submitting your revision, we need you to address these additional requirements.

Please ensure that your manuscript meets PLOS ONE's style requirements, including those for file naming. The PLOS ONE style templates can be found at 
<https://journals.plos.org/plosone/s/file?id=wjVg/PLOSOne_formatting_sample_main_body.pdf> and
<https://journals.plos.org/plosone/s/file?id=ba62/PLOSOne_formatting_sample_title_authors_affiliations.pdf>.

**RESPONSE: Thank you for this comment. I adhered to these additional requirements for PLOS ONE’s style requirements and file naming for the resubmission of the revised version of my manuscript.**

2. We note that you have indicated that there are restrictions to data sharing for this study. PLOS only allows data to be available upon request if there are legal or ethical restrictions on sharing data publicly. For more information on unacceptable data access restrictions, please see <http://journals.plos.org/plosone/s/data-availability#loc-unacceptable-data-access-restrictions>.

Before we proceed with your manuscript, please address the following prompts:

a) If there are ethical or legal restrictions on sharing a de-identified data set, please explain them in detail (e.g., data contain potentially identifying or sensitive patient information, data are owned by a third-party organization, etc.) and who has imposed them (e.g., a Research Ethics Committee or Institutional Review Board, etc.). Please also provide contact information for a data access committee, ethics committee, or other institutional body to which data requests may be sent.

b) If there are no restrictions, please upload the minimal anonymized data set necessary to replicate your study findings to a stable, public repository and provide us with the relevant URLs, DOIs, or accession numbers. For a list of recommended repositories, please see
<https://journals.plos.org/plosone/s/recommended-repositories>. You also have the option of uploading the data as Supporting Information files, but we would recommend depositing data directly to a data repository if possible.

We will update your Data Availability statement on your behalf to reflect the information you provide.

**RESPONSE: Thank you for this comment. This has been addressed as required by the journal and I provided the data as supplementary file in the resubmission.**

3. In the online submission form, you indicated that [Data are available on request from the author.]. 
All PLOS journals now require all data underlying the findings described in their manuscript to be freely available to other researchers, either 1. In a public repository, 2. Within the manuscript itself, or 3. Uploaded as supplementary information.
This policy applies to all data except where public deposition would breach compliance with the protocol approved by your research ethics board. If your data cannot be made publicly available for ethical or legal reasons (e.g., public availability would compromise patient privacy), please explain your reasons on resubmission and your exemption request will be escalated for approval.

**RESPONSE: Thank you for this comment. This has been revised as required by the journal and I provided the data as supplementary file in the resubmission.**

**Kind Note:** All line numbers are referred to in the manuscript with tracked changes.

**Reviewer 1 Comments**

Reviewer #1: Cross-Sectional Design Limitations
The use of a cross-sectional design limits the ability to infer causality between resilience and quality of life (QOL). While it is acknowledged that longitudinal designs may be more challenging to implement, it is important to clearly state this limitation and discuss how it affects the interpretation of the results. Additionally, the authors should consider suggesting longitudinal studies in the future research section to provide stronger evidence for causality.

**RESPONSE: Thank you for this comment. Limitation on using cross-sectional design has been clearly stated at the end of the ‘4. Discussion’ section. Please refer to Lines 436-438.** **Likewise, recommendation of conducting future longitudinal studies has been added in the ‘5. Implications and Recommendations’ section. Please refer to Lines 489-491.**

Convenience Sampling Bias
The use of convenience sampling raises concerns about the representativeness of the sample. This method can introduce selection bias, limiting the generalizability of the findings. The authors should acknowledge this limitation more explicitly in the manuscript and discuss its potential impact on the study's conclusions.

**RESPONSE: Thank you for this comment. This has been acknowledged at the end of the ‘4. Discussion’ section. Please refer to Lines 439-444.**

Self-Reported Data Concerns
Reliance on self-reported data can introduce response biases, such as social desirability bias and recall bias. The manuscript should discuss these limitations and consider suggesting the use of objective measures or triangulation with other data sources in future research to enhance the validity of the findings.

**RESPONSE: Thank you for this comment. This has been discussed in the limitations (Lines 444-445) and future research (Lines 491-494).**

Lack of Clear Topic Sentences
Several paragraphs lack clear and concise topic sentences, making it difficult for readers to follow the logical flow of the manuscript. Each paragraph should begin with a strong topic sentence that clearly introduces the main idea. The authors should revise these sections to improve readability and coherence.

**RESPONSE: Thank you for this comment. This has been addressed and revised throughout the manuscript, as suggested.**

Imprecise Reporting of Statistical Results
The statistical results reported in this study are sometimes imprecise. For example, stating the exact p-values (e.g. p = 0.032) instead of "<.05" would enhance transparency. Additionally, some tables lack sufficient details regarding the statistical tests used, making it challenging to fully interpret the results. The authors should provide more comprehensive details in the tables and the text.

**RESPONSE: Thank you for this comment. The exact *p* values have been added throughout the Results section. Please refer to Lines 258-310. Importantly, the following sentences below have been added for logical or clearer flow of presenting the results in Table 5. Please refer to Lines 279-281.**

*“The values corresponding to the percentage of the variance for each dimension of QOL are not presented in Table 5. However, the values presented in Table 5 are narratively described below.”*

Ethical Considerations and Anonymity
While the manuscript mentions that written informed consent was obtained, details on measures taken to ensure the anonymity and confidentiality of participants' data are also included. This information is crucial for maintaining ethical standards, and should be explicitly stated in the manuscript.

**RESPONSE: Thank you for this comment. This has been revised and corrected after further consideration that the respondents’ participation is kept confidential, and their responses are completely anonymous. Please refer to Lines 194-199.**

Risks to Participants
Potential risks to the participants were not adequately discussed. A thorough explanation of any risks involved in participation along with measures taken to mitigate these risks should be included to ensure adherence to ethical standards.

**RESPONSE: Thank you for this comment. This has been adequately addressed. Please refer to Lines 192-194.**

Validation of Measurement Tools
The manuscript mentions the validity and reliability of the Resilience Scale for Nurses and WHOQOL-BREF but lacks specific details on how these were confirmed in the current study population. Providing detailed psychometric properties specific to this sample would enhance the credibility of the instrument used.

**RESPONSE: Thank you for this comment. Information about the validity and reliability of the Resilience Scale for Nurses and WHOQOL-BREF have been added. Please refer to Lines 172-184.**

Translation Process
The translation process for any non-English version of the questionnaire used was not described. This is a critical omission because ensuring the validity of the translated instruments is essential. The authors should include a description of the translation and validation process.

**RESPONSE: Thank you for this comment. For clarification, there was no translation implemented in my study since the questionnaire was written in English language and the official business language used at KSUMC is English. The EFA and CFA for resilience instruments were from the previous study of Park et al. (2019).**

Linking Discussion to Research Questions
There are instances in which the discussion is not clearly linked to the study's aims and hypotheses. For example, the exploration of gender differences in QOL should be more explicitly connected to the research questions posed at the beginning of the study. The authors should ensure that all discussions are tied to the initial research objectives.

**RESPONSE: Thank you for this comment. This has been addressed in the ‘1.1 Aims’ section, where specific objectives were added. Please see Lines 105-113.**

Justification for Predictor Variables
The rationale for including specific predictor variables in the regression models is inadequately justified. The manuscript should provide a clear and compelling rationale for the inclusion of each predictor variable, as supported by relevant literature.

**RESPONSE: Thank you for this comment. This has been added in the ‘1. Introduction’ section. Please see Lines 73-91.**

Global Significance of Findings
While the focus on the Saudi Arabian context is justified, this manuscript should better articulate the global significance of the findings. Highlighting the broader implications and potential applications of the results would strengthen the contribution of the manuscript to the field.

**RESPONSE: Thank you for this comment. This has been addressed in the ‘6. Implications and Recommendations’ section. Please see Lines 465-494.**

Detailed Justification of Hypotheses
The introduction lacks detailed justification for the specific hypotheses tested. A more thorough explanation of the theoretical underpinnings and the empirical evidence guiding the hypotheses would provide a stronger foundation for this study.

**RESPONSE: Thank you for this comment. This has been added in the ‘1. Introduction’ section. Please see Lines 73-91.**

Discussion of Limitations
The Limitations section is too brief and does not adequately discuss how each limitation impacts the findings. A more detailed analysis of the study's limitations and suggestions for mitigating these issues in future research are necessary.

**RESPONSE: Thank you for this comment. This has been adequately addressed (Lines 434-447) and mitigating the limitation issues has been clearly discussed (Lines 489-494).**

Specific Future Research Recommendations
Recommendations for future research are generally general and lack specificity. Providing targeted and actionable suggestions based on the identified gaps and limitations is valuable.

**RESPONSE: Thank you for this comment. This has been addressed and emphasized in a separate section, ‘6. Implications and Recommendations’. Please refer to Lines 489-494.**

Assumptions of Multivariable Multiple Regression
The manuscript uses multivariable multiple regression appropriately, but lacks details on the assumptions checked before performing these analyses (e.g. multicollinearity and normality). Providing this information is crucial to assess the robustness of the statistical methods used.

**RESPONSE: Thank you for this comment. This has been addressed sufficiently in Lines 219-223.**

Avoiding "Impact" in the Title
The use of the term "impact" in the title is misleading, as the study's design does not allow causality to be established. A more accurate term would be "association" or "relationship.

**RESPONSE: Thank you for this comment. As suggested, I revised the title of my paper, *“Association of personal and professional factors, resilience and quality of life of registered nurses in a university medical city in the Kingdom of Saudi Arabia.”* The phrase ‘in a university medical city’ was also added based on the suggestion of another reviewer.**

Articulating Study's Importance
While this study addresses a significant topic, its importance could be better articulated in terms of its potential influence on policy and practice. The manuscript should emphasise how the findings can inform healthcare policies and interventions in more detail.

**RESPONSE: Thank you for this comment. This has been addressed and emphasized in a separate section, ‘6. Implications and Recommendations’. Please refer to Lines 465-474.**

Recommendation:
There are substantial areas that require improvement, particularly in methodology, reporting, and discussion. Therefore, we recommend that the manuscript be revised with major changes before it can be considered for publication. These revisions should address methodological weaknesses, enhance the clarity and coherence of the writing, and provide a more robust discussion and justification of the findings.

**RESPONSE: Thank you for this comment. I revised my manuscript based on this important recommendation as well as the valuable suggestions of Reviewers 3 and 4, especially in the Methods section (e.g., sample size calculation) and Discussion section (e.g., inclusion of more recent studies related to COVID-19 and additional comparison to studies conducted in Saudi Arabia.**

**Reviewer 2 Comments**

Reviewer #2: The manuscript is well written and it discussed an important topic. The introduction section outlines the important of the topic, the methods section addressed all required elements, the results section presents the findings in a readable manner

**RESPONSE: Thank you very much for this positive feedback. I am earnestly hoping that this will help to a favorable decision of the Academic Editor to the eventual acceptance of my manuscript for publication in PLoS ONE.**

**Reviewer 3 Comments**

Reviewer #3: hank you for giving me the opportunity to review this manuscript. The authors made a good effort in writing this study. However, there are some comments about the quality of the research and its presentation in a suitable way for the readers.

**RESPONSE: Thank you very much for this comment and hopeful that this may help the Academic Editor to decide on the eventual acceptance of my paper for publication.**

Please justify selecting nurses with experience of at least six months.

**RESPONSE: Thank you for this comment. The justification for selecting nurses with experience of at least six months has been added, completion of the probationary period in the hospital. Please refer to Lines 129-130.**

Add more information about the sample size calculation.

**RESPONSE: Thank you for this comment. Information about the sample size calculation has been added. Please refer to Lines 119-126. Correspondingly, I provided the response rate in the first part of the ‘3. Results’ section. Please see Line 228.**

The sample was selected from a single hospital; therefore, the title of the paper is supposed to reflect that instead of generalizing this to Saudi Arabia.

**RESPONSE: Thank you for this comment. The phrase ‘in a university medical city’ has been added in the title of my paper.**

Studies related to COVID-19 are out of date; however, the author may link the clinical implications of this study to more applicable lessons for any health crisis.

**RESPONSE: Thank you for this comment. Recent studies related to COVID-19 have been added. Please refer to Lines 320, 361-363, and 425-429. Additionally, the implications of this study have been emphasized in a separate section as ‘6. Implications and Recommendations’. Please refer to Lines 465-474.**

**Reviewer 4 Comments**

Reviewer #4: This study examines the resilience and quality of life (QOL) of nurses in Saudi Arabia during the COVID-19 pandemic. The topic is relevant given the significant impact the pandemic has had on healthcare workers globally. The study methodology appears sound, using validated scales and appropriate statistical analyses. The manuscript is organized and clearly written.

**RESPONSE: Thank you very much for this positive feedback. I hope this will help to a favorable decision of the Academic Editor to the eventual acceptance of the revised version of my manuscript.**

Abstract:
- The abstract is concise and provides a good overview of the study.

**RESPONSE: Thank you so much for this comment.**

- Add “cross-sectional” to the statement “in this quantitative study”.

**RESPONSE: Thank you for this comment. This has been added, as suggested.**

Methods
- “Ethical Considerations” section is too long. Some statements are redundant and not needed. For example, “The principle of justice also included the right to privacy. The respondents were not harmed mentally, emotionally, or in any other aspect”.

**RESPONSE: Thank you for this comment. These parts have been deleted, as suggested.**

- Always mention the full term of any abbreviation when first appears in the text. For example, “EFA and CFA” in line 118.

**RESPONSE: Thank you for this comment. The full terms of the following abbreviations, EFA and CFA have been provided. Likewise, full terms of other abbreviated terms in the manuscript have been mentioned where the term first appeared throughout the manuscript text.**

- Author stated, “Before the conduct of the regression analyses, the predictor variable nationality was dummy coded.” I think all categorical/binary predictors were dummy coded.

**RESPONSE: Thank you for this comment. This has been revised, as suggested. Please see Lines 215-217.**

Discussion:
- When comparing your results to prior studies, it is important to note if findings align with or differ from those reported from the same region such as: (<https://pubmed.ncbi.nlm.nih.gov/35983443>) who also examined quality of life and academic resilience in Saudi nursing.

**RESPONSE: Thank you for this comment and I have added this source as a good comparison of the results of my study to prior studies in the country. Please refer to Lines 373-376. In addition, I added comparison regarding the quality of life among healthcare workers across 19 countries in the Arab region, two years after the pandemic started (Ghazy et al., 2022). Please refer to Lines 361-363. Correspondingly, these citations have been updated with the numbering in the main text as well as in the ’References’ section.**

- The discussion is missing the discussion of potential confounders that may affect QOL of nurses other than those addressed in the current study. For example, occupational factors such as shift work and lifestyle factors such as sleep, diet, and stress have been reported from the same country:
o <https://pubmed.ncbi.nlm.nih.gov/26837403/>
o <https://pubmed.ncbi.nlm.nih.gov/38549756/>
**RESPONSE: Thank you for this comment. The following citations of Almajwal (2016) and Alghamdi et al. (2024) have been added, as suggested. Please refer to Lines 430-433. Correspondingly, these citations have been updated with the numbering in the main text as well as in the ’References’ section.**

- Conclusion section is very long. It can be shortened.

**RESPONSE: Thank you for this comment. The ‘5. Conclusions’ section has been revised and shortened, as suggested. Notably after reviewing the content of the second paragraph in the ‘Conclusions’ section, the second paragraph has been designated as a separate section, ‘6. Implications and Recommendations’ section. I am happy to take further feedback regarding this from the honorable reviewer.**

**CLOSING REPONSE: May the revisions I made on the revised version of my manuscript satisfy the Respectable Reviewers and Academic Editor. If not, kindly know that I am very willing to revise further so that my work will be eventually published in PLoS ONE journal. Thank you so much.**
